# Gas phase water-initiated droplet-assisted growth and shaping (DAGS) synthesis of poly(ethyl cyanoacrylate) (PECA) nanostructures

**DOI:** 10.1039/d5ra04467g

**Published:** 2025-09-04

**Authors:** Rabab Azizi, Stefan Seeger

**Affiliations:** a Department of Chemistry, University of Zurich Winterthurerstrasse 190 8057 Zurich Switzerland sseeger@chem.uzh.ch

## Abstract

The DAGS bottom-up method employs water nanodroplets as ‘templates’ for the fabrication of diverse 1D polymeric nanostructures. Herein, we successfully applied this approach in the gas phase to obtain poly(ethyl cyanoacrylate) nanofibres (PECA-NF) with a diameter ranging from 137 to 721 nm. Their 1D growth was unveiled using energy-dispersive X-ray spectroscopy (EDX).

## Abstract 2

This work highlights the versatility of the DAGS mechanism by showing its successful use in gas-phase fabrication of PECA nanofibres. Parameters like monomer concentration, relative humidity, and reactor capacity influence their 1D growth.

## Graphical abstract

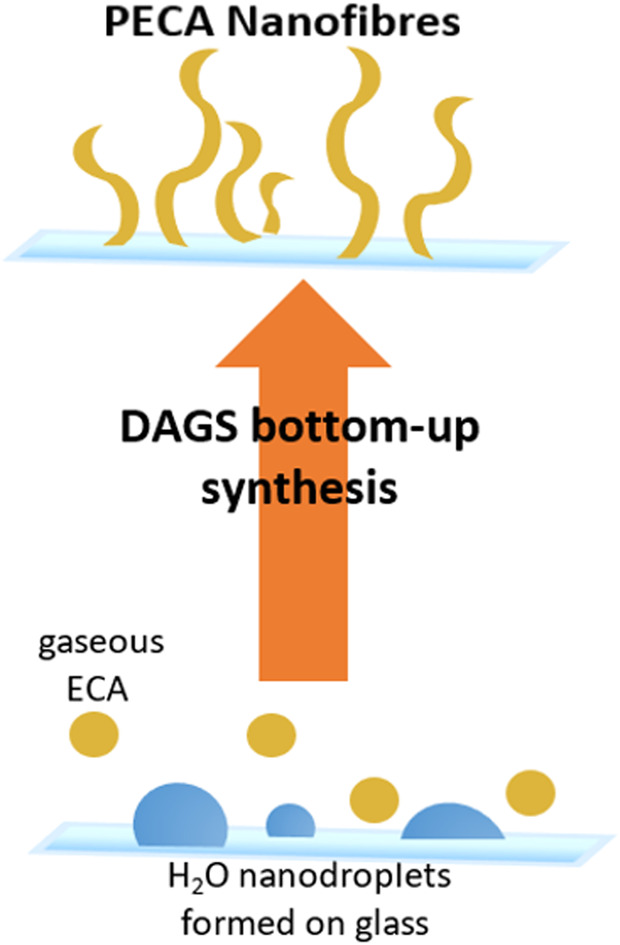

In the realm of nanotechnology, particularly when addressing the design of one-dimensional nanostructures two pivotal paradigms come to mind: namely, the bottom-up approach and the top-down approach.^1,2^ While the top-down strategy relies on breaking down a system into its single components to understand and manipulate it, the bottom-up approach offers a contrasting perspective by building systems from the molecular or atomic level.^3,4^ The bottom-up methodology is renowned for its design precision, efficient use of material, and capacity to achieve complex nanostructures with no waste of materials,^5^ in contrast to the top-down approach, which faces challenges like high material costs and difficulties to scale.^6^ The bottom-up techniques for the growth of polymeric 1D nanostructures include sol–gel synthesis,^7^ hydrothermal growth,^8^ self-assembly,^6,9^ and chemical vapour deposition.^5,8^ Notably, gas-phase synthesis has gained recognition as a versatile,^10^ inexpensive and scalable^11^ method for producing high-quality 1D nanomaterials. Within the gas-phase synthesis, the Droplet Growth and Shaping (DAGS) mechanism has proven to be an effective method for the fabrication of versatile polymeric 1D nanostructures ranging from silicone,^12^ mixed alumina–silicone^13^ 1D nanostructures to germanium oxide nanofilaments^14^ on both lab and pilot scales.^15^ Although this mechanism has also been used in solution,^16,17^ it is more established in the gas phase (Fig. 1) owing to its straightforward and scalable reaction setup, as well as minimized hazards resulting from the elimination of solvent use—an important advantage, particularly at a large scale.^15^ As its name indicates, the droplet-assisted growth and shaping mechanism (DAGS) is a bottom-up method in which the deliberately generated water nanodroplets on a substrate surface participate in the growth and shaping the 1D nanostructures *via* spontaneous polymerisation between the corresponding monomer and water. The 1D growth is sustained by polymer insolubility in water. In the gas phase, the choice of monomer is primarily dictated by its volatility where relative humidity (RH) and monomer concentration play crucial roles in determining the final morphology.^12–14^

**Fig. 1:**
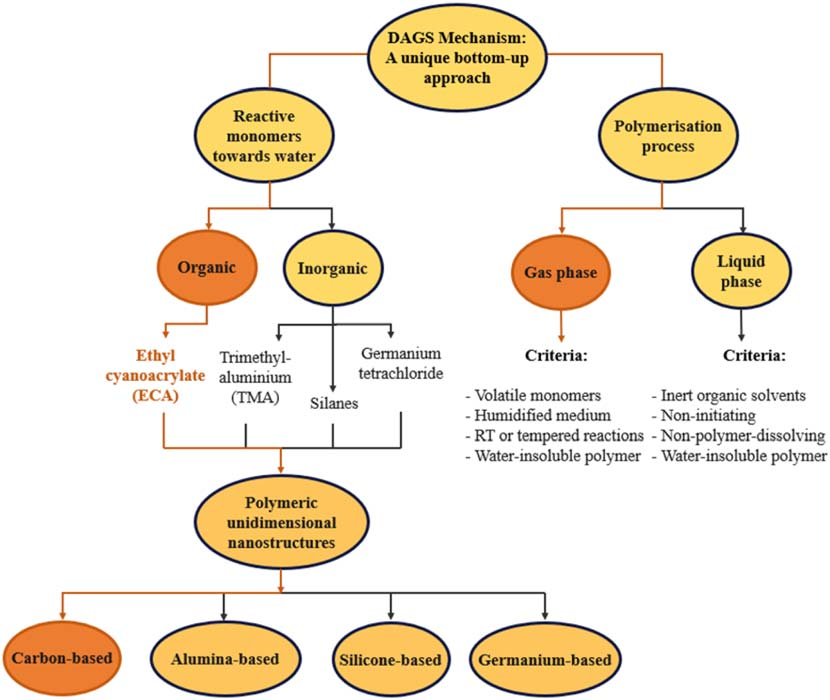
Chart depicting the polymerisation processes and monomers used in the DAGS bottom-up synthesis for the fabrication of 1D polymeric nanostructures. The orange arrows indicate the strategy we used to produce the PECA-NF in this study.

PECA nanofibres have been detected on the crests of latent fingerprints^18^ after the fuming process, a technique routinely employed in forensic science^19–23^ and typically performed at a relative humidity of 80%.^24^ Such fibrous nanostructures are believed to be initiated by sweat and sebum present in the skin.^25^ Since this discovery, several research groups have drawn inspiration from this phenomena, fabricating PECA nanofibres using various initiators.^26,27^ In this study, we present an alternative approach for the fabrication of PECA nanofibres (PECA-NF) in the gas phase *via* the DAGS mechanism by systematically investigating the effect of critical parameters – specifically, ECA mole amount, relative humidity and reactor capacity – on the morphological characteristics of PECA nanostructures. The 1D growth of the obtained PECA-NF was demystified through EDX analysis, providing robust evidence of our mechanism. By extending the DAGS mechanism to the gas-phase synthesis of PECA, we aim to deepen our understanding of the formation of not only inorganic 1D nanostructures but also the ones with a carbonic backbone and thus, contribute to valuable insights into the design and fabrication of versatile advanced 1D nanostructures. For this purpose, the gas-phase polymerisation of ECA was carried out in two borosilicate reactors of different capacities: approximately 120 mL (small reactor, SR) and 673 mL (large reactor, LR). Both reactors were heated using a silicone bath at 50 °C. After placing the glass substrate in the reactor, the relative humidity was carefully regulated using a nitrogen stream. Finally, ECA was injected and allowed to polymerise for 1.5–2 hours. Three different mole quantities of ECA (0.85, 1.70, and 3.39 mmol, corresponding to 100, 200, and 400 μL) were polymerised at RH 30%, 51%, 69%, and 88%, each with a maximal variation of *ca.* 2%. Samples are named as S-(*z*)m(*y*) or L-(*z*)m(*y*), where “S” and “L” imply small and large reactor, respectively. The number (*z*) represents the ECA volume and *m* stands for microlitres. Whereas (*y*) denotes the value of relative humidity during polymerisation.

Fig. 2 presents the SEM images of various PECA nanostructures (PECA-NS) obtained from the polymerisation of 0.85 to 3.39 mmol of ECA at relative humidity values ranging from 30% to 88% in both the small reactor (Fig. 2a–l) and the large reactor (Fig. 2m–x). The resulting PECA nanostructures include quasi-PECA films—primarily observed at low to moderate humidity (RH 30–69%) and low monomer mole amounts (Fig. 2b, m and o). Some of the PECA films exhibited rough surfaces (Fig. 2e and k) or, particularly at higher humidity (69–88% RH), display local patterns or dispersed, popcorn-like nanostructures (Fig. 2g, l, p, s, w and x). Polymerisation of high monomer mole amounts and low humidity (RH 30%) in both reactors resulted in dispersed PECA islands (Fig. 2i, q, and u). PECA fibrous nanostructures (PECA-FNS) also appeared, either as surface-spread nanofibres (Fig. 2a and j) or as nanofibre bundles (Fig. 2c, t, and v).

**Fig. 2:**
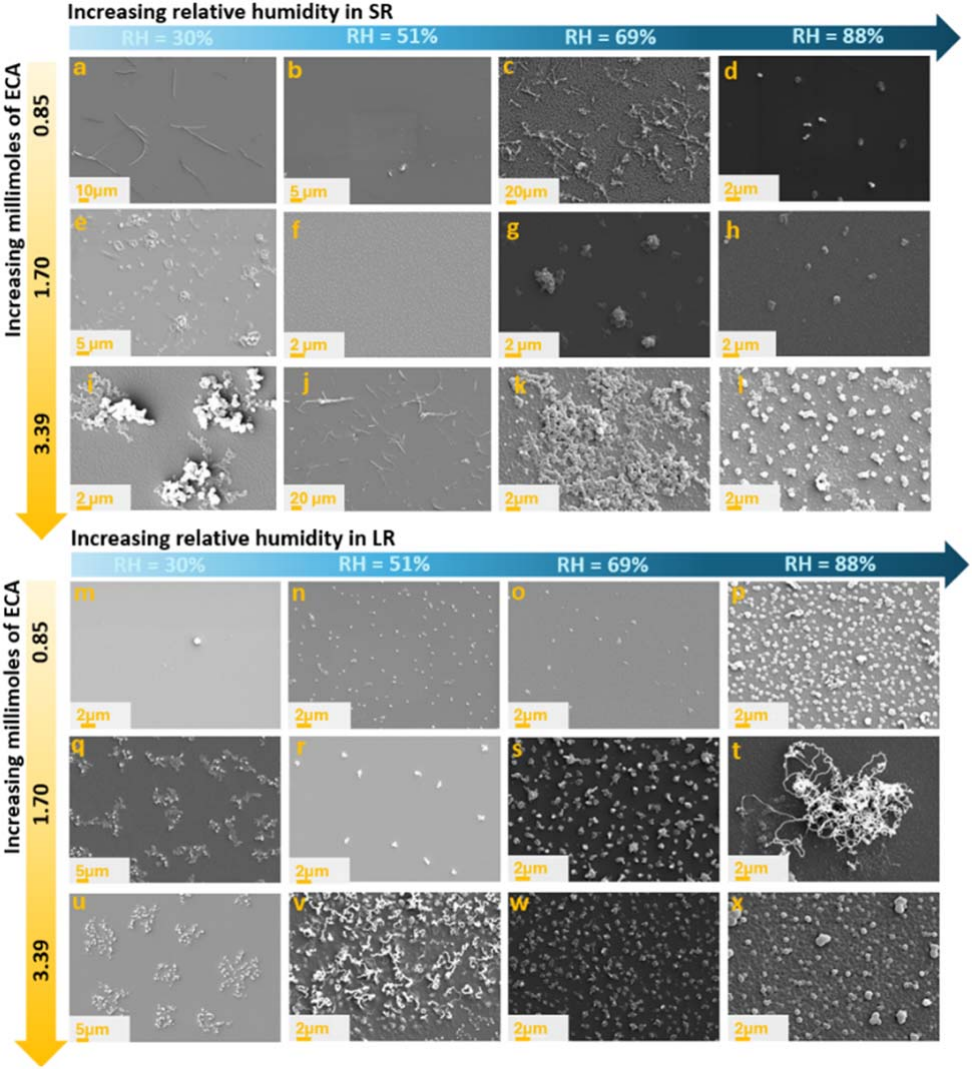
SEM images of PECA nanostructures obtained during the polymerisation of different ECA mole amounts while variating RH in the small reactor (a–l) and large reactor (m–x): (a) S-100m30; (b) S-100m51; (c) S-100m69; (d) S-100m88; (e) S-200m30; (f) S-200m51; (g) S-200m69; (h) S-200m88; (i) S-400m30; (j) S-400m51; (k) S-400m69; (l) S-400m88; (m) L-100m30; (n) L-100m51; (o) L-100m69; (p) L-100m88; (q) L-200m30; (r) L-200m51; (s) L-200m69; (t) L-200m88; (u) L-400m30; (v) L-400m51; (w) L-400m69; (x) L-400m88.

Close-up SEM images of these PECA-FNS, along with their average diameter, is shown below in Fig. 3.

**Fig. 3:**
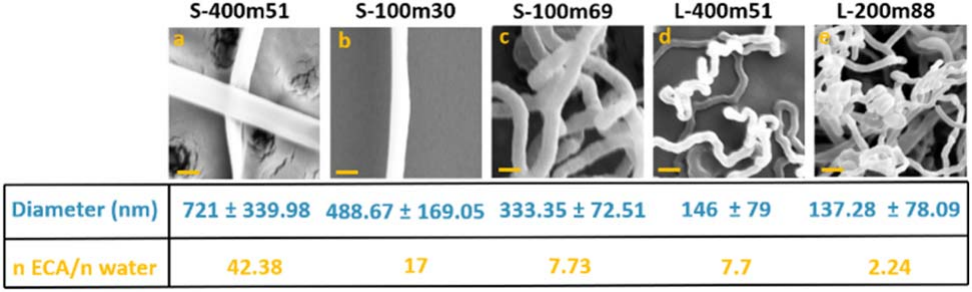
Magnified SEM images of PECA-FNS and their measured average diameter of the PECA-FN along with their respective monomer-to-water ratio: (a) sample S-400m51; (b) sample S-100-30; (c) S-100m69; (d) sample L-400m51 and (e) sample L-200m88. The scale bar represents 400 nm.

It is noticeable that a greater number of fibrous PECA-FNS were observed in the small reactor rather than the large one. This could be attributed to the fact that, under identical relative humidity and temperature conditions, the larger reactor retains a greater absolute amount of water vapour. The increased number of water molecules provides more initiation sites on the substrate but also raises the likelihood of termination reactions. This may account for the smaller diameters of PECA nanofibres synthesized in the large reactor—137.28 ± 78.09 nm (sample L-200m88) and 146 ± 79 nm (sample L-400m51)—compared to those produced in the small reactor, where diameters vary between 333.35 ± 72.1 nm and 721 ± 339.89 nm. Moreover, increasing the calculated monomer-to-initiator (*n*_ECA_/*n*_water_) ratio (SI eqn (1)–(3)) led to thicker nanofibres as presented in Fig. 3, likely because longer polymer chains are formed, resulting in fewer active sites and a lower chance of termination. Interestingly, in the larger reactor, nanofibre diameters stayed fairly consistent even when this ratio changed. This suggests that the growth of PECA nanofibres may depend not only on polymerisation kinetics, but also on complex surface phenomena, such as the dynamic of water in the reaction vessel as well as the interaction of water nanodroplets with the substrate, which likely dictate their shape. To gain a deeper understanding of the 1D growth of the obtained PECA-NF, we exposed a glass slide previously coated with hydroxyapatite (HA) to 1.70 mmol of ECA vapour at a relative humidity of ∼89 ± 1.2%. The objective was to ‘map’ the unidimensional growth of PECA *via* elemental analysis of phosphorus, which could subsequently be detected using EDX. Scanning electron microscopy (SEM) images confirmed the presence of PECA-NF and EDX analysis was carried out at 4 various sites to collect signals from phosphorus, as illustrated below in Fig. 4.

**Fig. 4:**
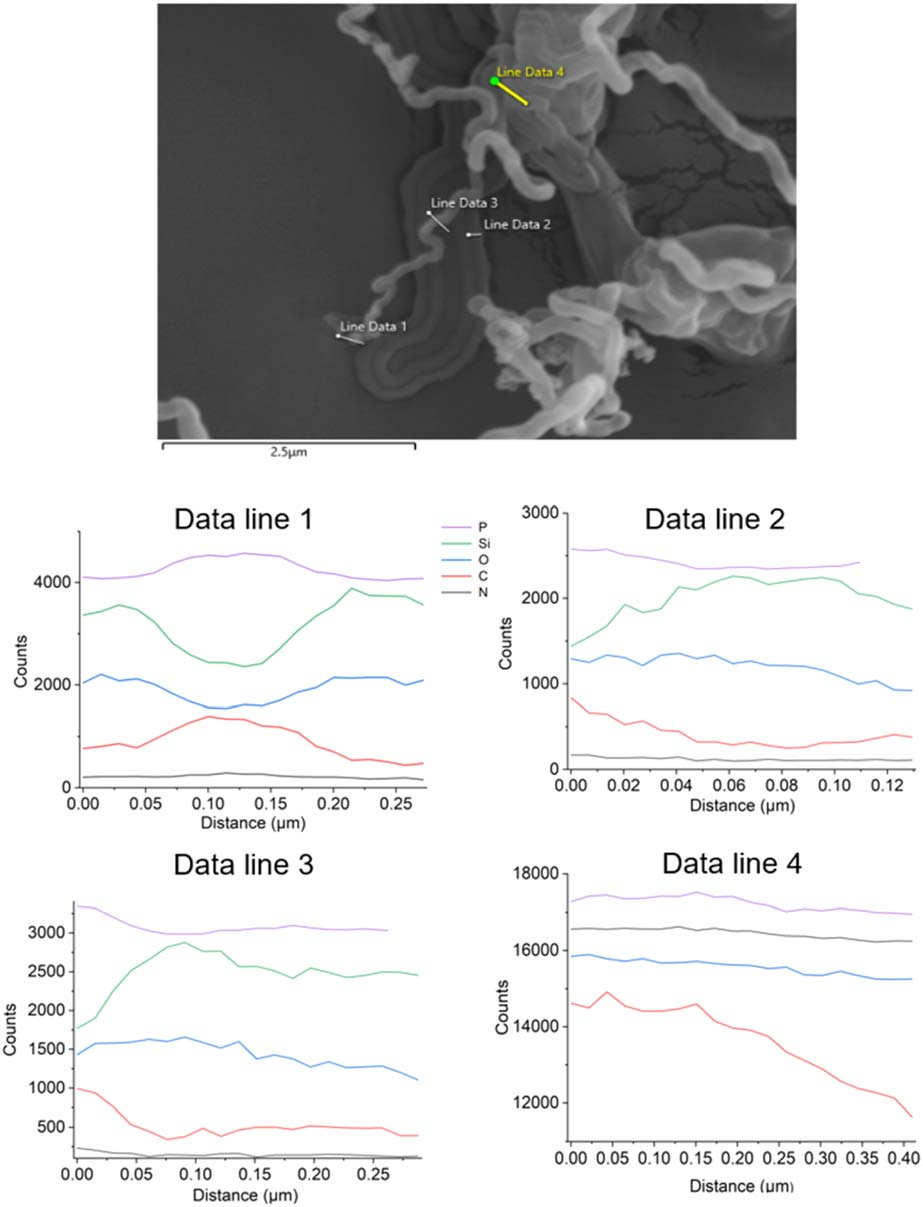
SEM image of PECA nanofibres formed on hydroxyapatite-coated glass slides (top), with EDX elemental analysis at four sites (bottom): scan line 1-tip of the nanofibre; scan line 2-PECA film; scan line 3-middle of the nanofibre; and scan line 4-PECA clump. Purple represents phosphorus, green silicon, blue oxygen, red carbon, and black nitrogen.

EDX analysis revealed that phosphorus was detected exclusively at the tip of the nanofibre (see Fig. 4 Data line 1). Furthermore, an increase in carbon counts, accompanied by a decrease in both silicon and oxygen, indicates that the analysed fibre is most likely composed of PECA. In Fig. 4, Data line 2 – collected from the formed PECA film – shows relatively constant phosphorus counts, although these are lower than those observed in Data line 1. This indicates that phosphorus is still present in the PECA film, but in smaller amounts at the base of the coating compared to the tip of the nanofibre, which is to be expected since the glass slide was coated with HA. Additionally, the decrease in carbon counts alongside a simultaneous increase in silicon and oxygen signals suggests that there is less PECA present compared to the fibre. Examining Data line 3, which was collected from the middle of the nanofibre, one can observe that the concentration of phosphorus is higher than in Data line 2, although the phosphorus counts slightly decrease along the fibre diameter. Nevertheless, there is more phosphorus present than in the film (Data line 2), implying that HA was transported upward from the coating base. The phosphorus counts remain, however, highest in Data line 1, indicating that the greatest amount of phosphorus is located at the tip, and thus, more HA has been transported to the top. The results from scan line 4 show the highest levels of both carbon and phosphorus on a sort of a polymer clump. This could be due to the formation of a HA clump during the dip coating on which ECA was later polymerised. Alternatively, the coalescence of numerous water nanodroplets, which swelled an increased amounts of HA on the surface may have induced a local imbalance between the initiators (water–hydroxyapatite) and the ECA monomer, leading to the formation of an irregular polymer structure. Furthermore, the development of a PECA agglomerate rather than well-defined nanofibres could be attributed to premature termination of the anionic polymerisation, potentially caused by the presence of gaseous water molecules during the propagation of the polymer chains on that site. Despite these possible surface ‘anomalies’ the data from scan lines 1–3 align perfectly with the DAGS mechanism. Here, water nanodroplets act as confined ‘templates’ for the growth of one-dimensional polymer nanostructures evidenced by the highest phosphorus EDX signals on the tip of the formed PECA nanofibre. The 1D growth of the PECA-NF *via* the DAGS approach when using HA as a ‘tracing agent’ can be illustrated as follows in Fig. 5.

**Fig. 5:**
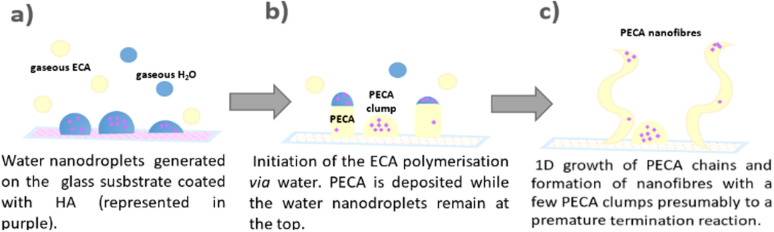
Illustration of the 1D growth of PECA-NF *via* the DAGS mechanism which was unveiled when using HA as a ‘tracing agent’: (a) the water nanodroplets are formed on the surface coated with HA (purple dots); (b) the polymerisation of ECA is initiated by water and eventually, by a few HA molecules. The water insoluble PECA is deposited. The water nanodroplets with solubilised HA remain on the top; (c) 1D growth of the PECA macromolecules and formation of PECA-NF.

First, humid nitrogen is introduced into the reaction chamber, forming water nanodroplets swelling locally HA on the surface (Fig. 5a). These represent initiation sites on which volatilised ECA polymerises into a water-insoluble polymer. PECA deposits at the bottom (Fig. 5b). As the PECA chains propagate, the water droplets with solubilised HA remain at the tip, guiding the continued 1D growth resulting in PECA-NF. A premature termination of the ECA polymerisation on a few sites on the surface lead to PECA clumps (Fig. 5c).

In conclusion, we have developed a novel gas-phase DAGS approach for the synthesis of PECA NF, by tuning relative humidity and reactor capacity. SEM imaging shows the formation of fibrous nanostructures with diameters ranging from *ca.* 137 to 721 nm, while their 1D growth *via* the DAGS mechanism is confirmed through EDX analysis. This work not only expands the scope of DAGS to organic polymers such as PECA but also emphasises the critical role of water nanodroplet dynamics and surface interaction in the formation of such nanostructures. Thus, collectively, our findings lay the groundwork for future mechanistic investigations and open new avenues for the DAGS-initiated polymerisation to target a broader and more versatile spectrum of 1D nanomaterials.

## Conflicts of interest

The authors have no conflicts of interest to declare.

## Supplementary Material

RA-015-D5RA04467G-s001

## Data Availability

All the raw data used in this article can be accessed from OSF repository at OSF|Gas-phase DAGS synthesis of PECA-NF (https://osf.io/uwdvz/). Supplementary information is available. See DOI: https://doi.org/10.1039/d5ra04467g.
