# Supplementary material for: Gas phase water-initiated droplet-assisted growth and shaping (DAGS) synthesis of poly(ethyl cyanoacrylate) (PECA) nanostructures
Source: RSC Adv. 2025 Sep 4;15(38):31763–6. doi: 10.1039/d5ra04467g (PMC12409712; doi:10.1039/d5ra04467g)
Supplement: RA-015-D5RA04467G-s001 [file RA-015-D5RA04467G-s001.pdf]

Supplementary Information (SI) for RSC Advances.
This journal is © The Royal Society of Chemistry 2025

Supporting Information

Gas phase water-initiated droplet-assisted growth and shaping (DAGS) synthesis of poly (ethyl cyanoacrylate) (PECA) nanostructures

Rabab Azizi, [a] Stefan Seeger [a*]

Table of Contents

1 Materials 2
2 Methods 2
2.1 Substrate pretreatment 2
2.2 The DAGS synthesis of PECA nanostructures 2
2.3 Magnified SEM Images of the PECA-FNS 3
2.3.1 Calculation of n_{ECA}/n_{water} ratio and its correlation with PECA-NF diameter 5
2.4 FT-IR measurement of the obtained PECA-NF 7
2.5 Coating with hydroxyapatite (tracing agent) and EDX analysis 8

1 Materials

Ethyl cyanoacrylate (ECA) and hydroxyapatite (HA) were purchased from Merck (Switzerland) and Deconex 11 Universal was obtained from Borer Chemie (Switzerland). Glass slides (25 mm× 60 mm ×1 mm) were obtained from Epredia-Menzel and were used as a substrate. MQ-water was generated from

2 Methods

2.1 Substrate pretreatment

The glass substrates were pretreated by immersion in a 10% v/v Deconnex 11 Universal aqueous solution, followed by sonication in an Elmasonic X-tra 70 H (Elma Schmidbauer GmbH, Germany) ultrasonic bath using the soft setting for 30 minutes at 50 °C. Subsequently, the substrates were thoroughly rinsed with ultrapure water and dried in one direction using a nitrogen gun.

2.2 The DAGS synthesis of PECA nanostructures

Gas-phase polymerisation of ECA was carried out in two cylindrical borosilicate glass reactors, referred to as the large reactor (LR) and small reactor (SR), with approximate capacities of 673 mL (diameter ≈ 82 mm) and 120 mL (diameter ≈ 35 mm), respectively. Each reactor is equipped with a nitrogen inlet and outlet, a septum, and a thermometer opening. A clean glass hour was put at the reactor's bottom to collect the later injected ECA monomer, whilst a permeable custom-made plastic sample holder was placed in the upper third of each reactor. To ensure homogeneous heat distribution, the reactor was immersed in a silicone bath maintained at 50°C with constant stirring at 300 rpm. The reactor temperature was monitored via an inserted thermometer indicating a temperature of 25±1°C.

The relative humidity (RH) within the reactor was introduced using two nitrogen sources: One directly from the lab dry nitrogen supply (DN_2) and another directed through a Woolf's flask containing Milli-Q water to generate wet nitrogen (WN_2). Prior to the polymerisation, the clean pretreated glass is placed in the reactor. For the large reactor the whole pretreated glass slide was used while for the small one the glass slide was cut in a dimension of approximately L × w = 25 mm× 15 mm. The reactor is sealed, and nitrogen is flushed inside the reactor with the desired humidity for 1 -1.5 hours. The RH was continuously monitored using a EE23 hygrometer (E+E Elektronik). Finally, the nitrogen inlet and outlet valves were closed and the ECA monomer was injected into the reactor.

The polymerisation was allowed to proceed for 1.5-2 hours, after which the substrate was taken out and stored in a polypropylene slide mailer for subsequent analysis. The polymerisation was carried out using different molar amounts of ECA, namely 0.85, 1.70 and 3.39 mmol were polymerised at relative humidities of ~30%, 51%, 69%, and 88% with a maximal variation of up to 2% for each. The sample nomenclature is structured as S-(z)m(y) or L-(z)m(y), where the initial letter denotes the reactor type (S for small reactor, L for large reactor). The first number (z) indicates the injected volume of ECA in microlitres (m), and the final number (y) specifies the value of relative humidity at which the

polymerisation was performed. For example, samples coated with 0.85 mmol ECA at 30% relative humidity in SR and with 3.39 mmol ECA at 88% relative humidity in LR are designated as S-100m30 and L-400m88, respectively. The setup used for the DAGS water-initiated ECA in the gas phase is depicted in figure S1:

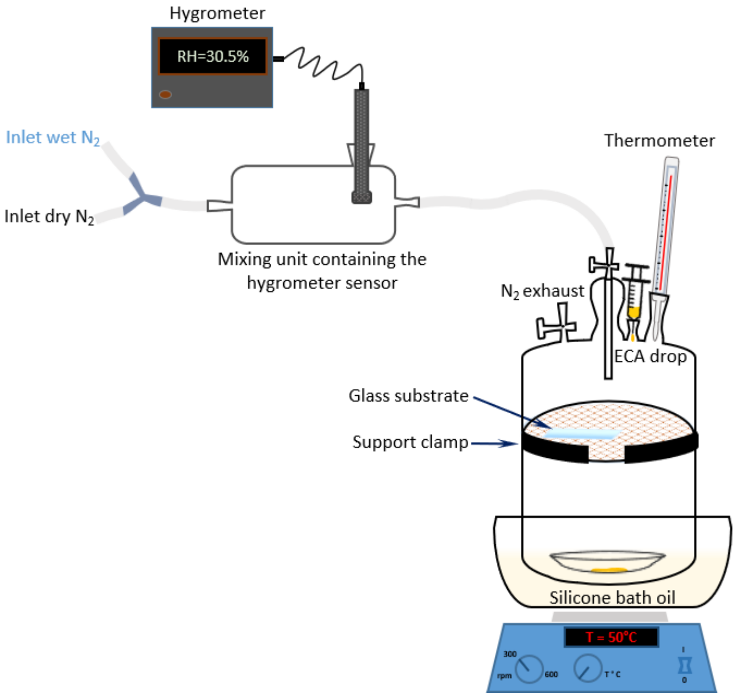

S 1. Scheme of the experimental set-up used for the polymerisation of ECA in the gas phase *via* the DAGS mechanism.

2.3 Magnified SEM Images of the PECA-FNS

SEM images were obtained using a Gemini 450 microscope at 10 kV using an in-chamber Everhart-Thornley type SE detector. Prior to the SEM analysis all samples were sputter coated with a 5 nm of platinum using a Safematic CCU-010 sputter coater (Switzerland). The magnified SEM top views images of the formed PECA-FNS can be found below in figure S2:

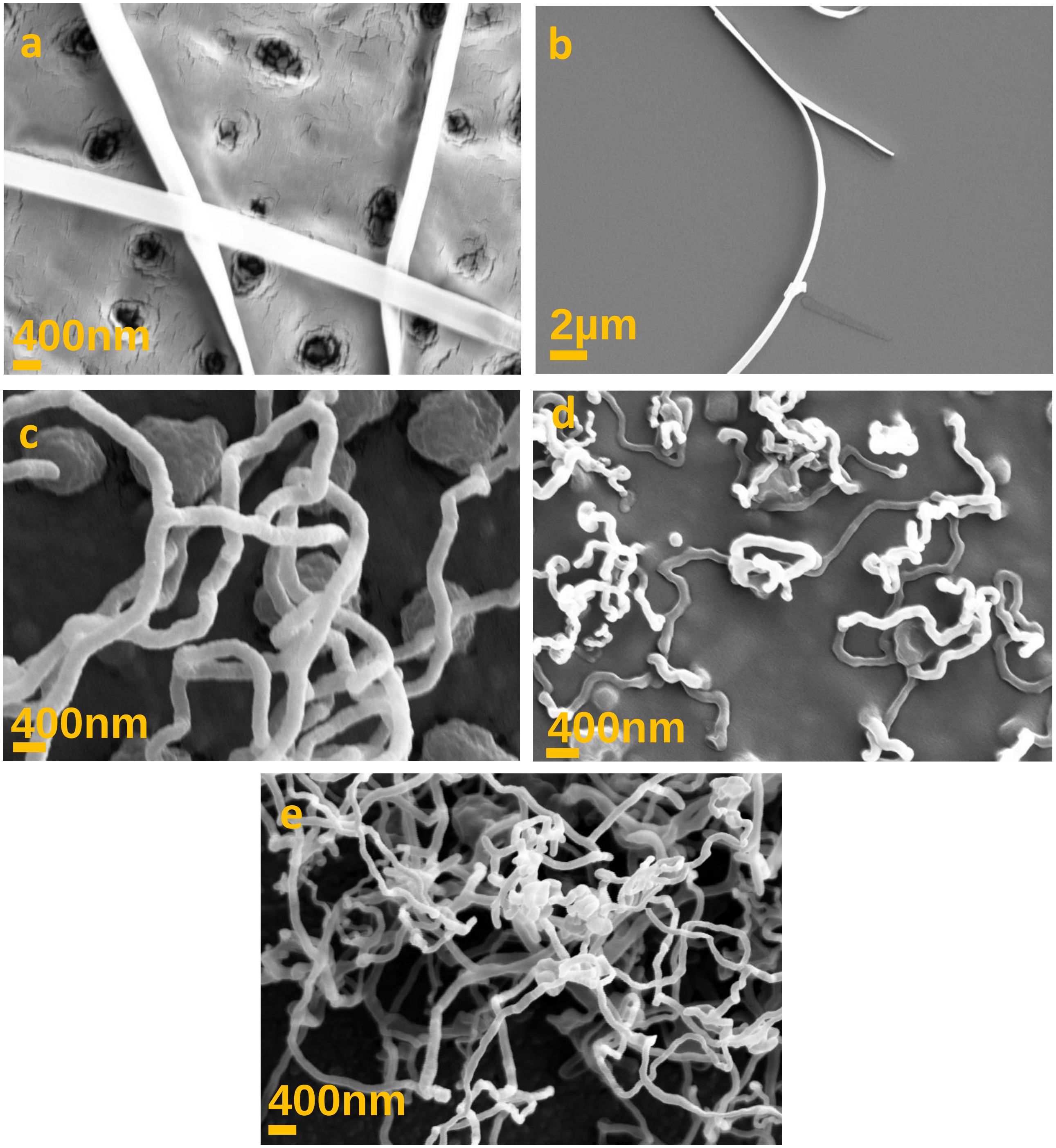

S 2. SEM top-view images of the obtained PECA-FNS: a) sample S-400m51; b) sample S-100-30; c) S-100m69; d) sample L-400m51 and e) sample L-200m88.

The SEM profile view the sample L-200m88 reveals more nest-like PECA-NF as shown below in S3:

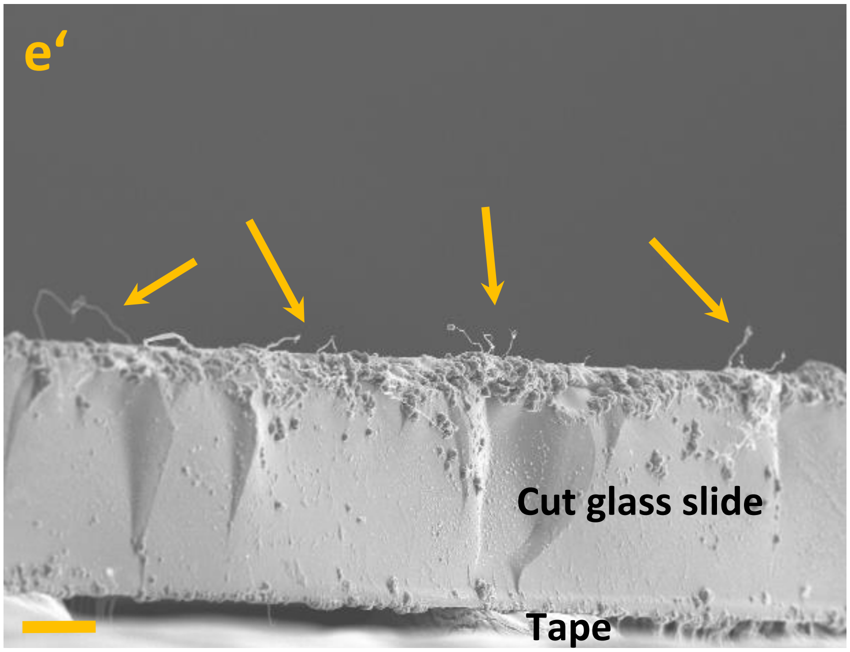

S 3. SEM profile-view of sample L-200m88: The orange arrows show more 'nests' of PECA- NF on the surface. The scale bar corresponds to 50 µm.

2.3.1 Calculation of n $_{ECA}$/n $_{water}$ ratio and its correlation with PECA-NF diameter

The ECA monomer to initiator (water) ratio can be expressed as:

$$r = \frac{n_{ECA}}{n_{Water}} \qquad \text{(Eq.1)}$$

The molar amount of water can be calculated from the relative humidity using the following equation:

$$P_{water} = RH \times P_{sat} \qquad \text{(Eq.2)}$$

Where P_{water} is the pressure of water, RH the relative humidity and P_{sat} the saturated water vapour which is 3.17 kPa at a temperature of 25°C.

Assuming that the water vapour behaves as an ideal gas, n_{water} can be deduced as follows:

$$n_{Water} = \frac{P_{water} \times V_{reactor}}{\mathbf{R} \times T} \quad \text{(Eq.3)}$$

Where $V_{reactor}$ is the volume of the corresponding reactor (0.12L for SR and 0.673L for LR). Whereas R= 8.314 L·kPa·K^{-1}·mol^{-1} is the gas constant and T the measured temperature inside the reactor which corresponds to 25 °C. Table 1 summarises the calculated values of P_{water}, n_{water} and r for the obtained PECA-NF in both SR and LR.

Table 1. Showing the calculate P_{water}, n_{water} and the monomer to initiator ratio r. The results show an increase in the PECA-NF diameter with increasing r in SR.

Sample	P_{water} (kPa)	n_{water} (mmol)	n_{ECA} (mmol)	r	PECA-NF diameter (nm)
S-100m30	0.95	0.05	0.85	17.00	488.67
S-100m69	2.19	0.11	0.85	7.73	333.35
S-400m51	1.62	0.08	3.39	42.38	721.00
L-200m88	2.79	0.76	1.7	2.24	137.28
L-400m51	1.62	0.44	3.39	7.7	146.00

2.4 FT-IR measurement of the obtained PECA-NF

IR measurements were performed using a vacuum-sealed ATR-FT-IR VERTEX 70v vacuum spectrometer within a spectral range of 3000 to 300 cm^{-1} and a resolution of 4 cm^{-1}. The presence of PECA nanofibres was confirmed specifically *via* the C=O absorption band around 1747-1749 cm^{-1}, which indicates the presence of PECA on the coated glass (S9).

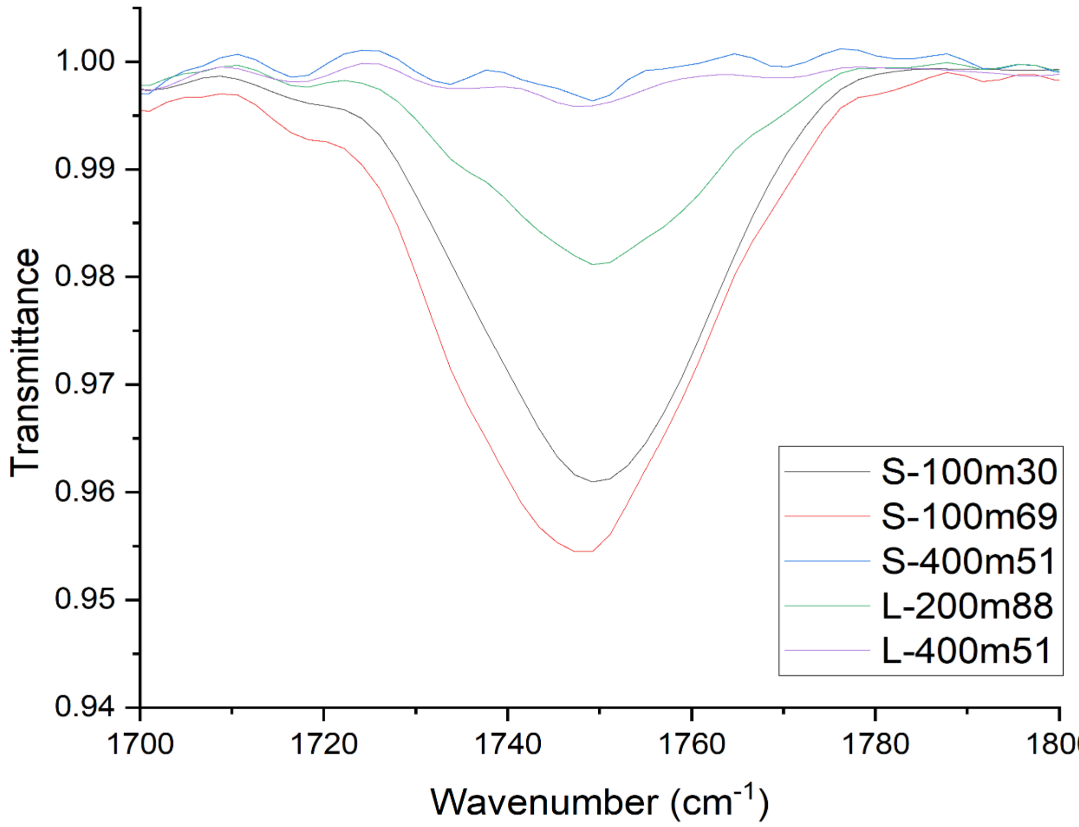

S 4. FT-IR absorption band of C=O indicated the presence of PECA-NF on samples coated in SR and LR at different RH.: beige corresponds to the sample S-100m30, red to S-100m69, green to L-200m88 and purple to L-400m51.

2.5 Coating with hydroxyapatite (tracing agent) and EDX analysis

A pre-treated glass slide was dip-coated overnight in approximately 42 mL of a 36 mg/L hydroxyapatite (HA) solution. Afterwards, the slide was dried with a nitrogen gun and then placed in a large reactor heated using a silicone bath at 50 °C. The slide was subsequently exposed to the vapours of 1.77 mmol of ECA under high relative humidity (RH = 89 ± 1.2%) to enable a maximal dissolution of HA on the surface during polymerisation. The sample was sputter-coated with a 5 nm platinum layer, and elemental analysis was performed using an integrated X-MAX80 X-ray detector within the Gemini 450 SEM device. Data were acquired at a working distance of 12.6 mm and processed with AZtec Advanced software (Oxford). The SEM image of the PECA-NF obtained with the HA-'tracer' can be seen on S 10.

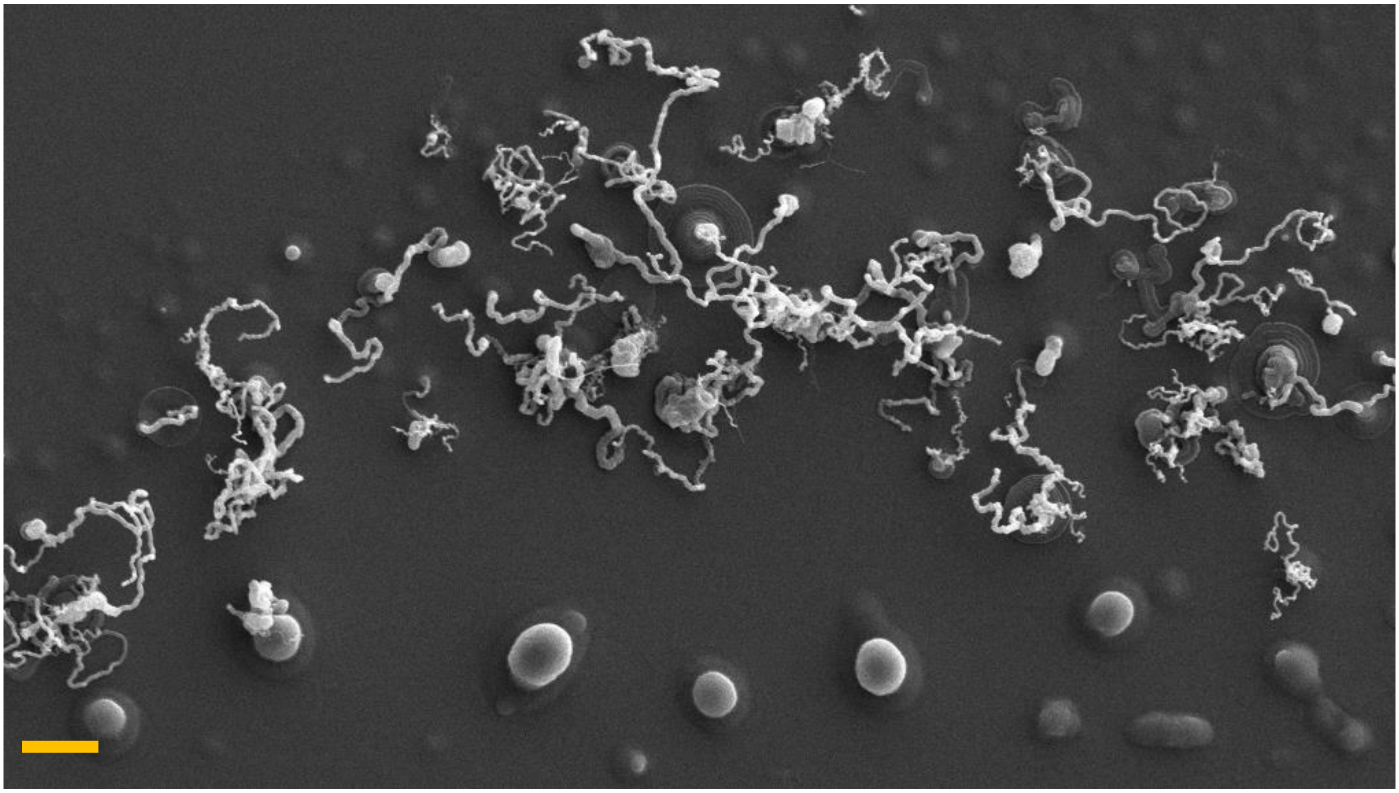

S 5. SEM image of PECA-NF formed on the HA-coated glass slide during the polymerisation 1.77 mmol ECA and 89% relative humidity. The surface displays also some coating anomalies i.e. PECA bumps thought to be caused by a premature termination of the polymerisation. The scale bar is 5µm.
